# TLR Signaling Beyond Immunity: Emerging Roles in Cell Migration, Metabolism, Development, and Host–Microbiome Crosstalk

**DOI:** 10.3390/biology15191778

**Published:** 2026-10-07

**Authors:** Li Liu, Herman P. Spaink

**Affiliations:** Institute of Biology Leiden, Animal Science and Health, Leiden University, Einsteinweg 55, 2333 CC Leiden, The Netherlands; liliu123096@hotmail.com

**Keywords:** TLR signaling, immunometabolism, development, microbiome, host–microbe interactions, zebrafish

## Abstract

Recent findings support a broader view of TLR signaling as an integrative signaling hub that links immunity with cell migration, metabolism, development, and host–microbiome crosstalk. In cell migration, TLRs exert context-dependent effects through chemokine receptor regulation, adhesion and cytoskeletal modulation, and indirect cytokine-mediated signaling. In metabolism, TLRs orchestrate insulin resistance, obesity, and glucose homeostasis in a receptor- and tissue-specific manner. In tissue injury and development, TLR signaling exerts dual roles; it is detrimental in ischemia–reperfusion injury, but beneficial in vascular remodeling and neurogenesis. We then examine the bidirectional interplay between TLRs and the gut microbiome, where TLRs shape microbial community structure and intestinal immune homeostasis. Finally, we survey the therapeutic landscape of TLR modulators, highlighting that while TLR agonists are predominantly explored in oncology, TLR antagonists show promise in autoimmune diseases such as inflammatory bowel disease. Special attention is given to insights from organoid, rodent and zebrafish models, which have provided powerful in vitro and in vivo evidence for multiple roles of TLR signaling under both physiological and pathological conditions. A deeper understanding of these non-canonical functions will be essential for developing next-generation therapeutic strategies that selectively modulate TLR signaling in infectious, inflammatory, metabolic, and microbiome-associated diseases.

## Simple Summary

Toll-like receptors (TLRs) are evolutionarily conserved pattern recognition receptors best known for their roles in innate immune defense against microbial pathogens. However, accumulating evidence has revealed that TLR signaling extends far beyond classical immunity to regulate diverse systemic processes, including cell migration, metabolism, development, and host–microbiome crosstalk. This review integrates recent advances in understanding the non-canonical functions of TLR signaling, with particular emphasis on insights gained from zebrafish, organoid and rodent models.

## 1. Introduction

Host innate immunity serves as the first line of defense against invading pathogens, providing rapid and broad recognition of conserved microbial structures before the onset of adaptive immune responses [1]. Central to this system are evolutionarily conserved pattern recognition receptors (PRRs), which detect pathogen-associated molecular patterns (PAMPs) derived from invading pathogens and host-derived damage-associated molecular patterns (DAMPs) released from damaged cells [2]. Among the best-characterized PRRs are Toll-like receptors (TLRs), a family of type I transmembrane proteins that are evolutionarily conserved from invertebrates to vertebrates [2]. TLRs are expressed on the plasma membrane or endosomal compartments of immune and non-immune cells, enabling the recognition of a wide range of ligands [3]. Upon ligand binding, TLRs recruit adaptor proteins such as myeloid differentiation primary response 88 (MyD88) or Toll/interleukin-1 receptor (TIR) domain-containing adaptor inducing interferon-β (TRIF) to activate downstream transcription factors, thereby orchestrating inflammation, antimicrobial responses and tissue repair [4,5,6].

The mammalian TLR family comprises ten functional members (TLR1-TLR10) in humans and twelve (TLR1-TLR9, TLR11-TLR13) in mice, each recognizing distinct PAMPs and DAMPs [7]. PAMPs are conserved microbial structures derived from invading pathogens. Cell surface TLRs (TLR1, TLR2, TLR4, TLR5, TLR6, and TLR10) primarily recognize microbial membrane components such as lipoproteins, lipopolysaccharide (LPS), and flagellin, whereas endosomal TLRs (TLR3, TLR7, TLR8, and TLR9) sense nucleic acids derived from viruses and bacteria [8]. DAMPs, in contrast, are endogenous molecules released from damaged or stressed cells, such as high-mobility group box 1 (HMGB1), heat shock proteins (HSPs), saturated fatty acids, and extracellular matrix fragments like hyaluronan and fibrinogen [9]. While PAMPs signal the presence of infection, DAMPs alert the host to tissue injury, and both classes of ligands activate TLRs to initiate inflammatory responses. The size of hyaluronan appears to determine responsiveness by TLRs [10,11]. In addition to their well-established functions in recognizing pathogens and cellular damage, TLRs have emerged as key modulators of tissue homeostasis, metabolic processes, and the regulation of symbiotic host–microbiome interactions, underscoring their importance in both health and disease contexts [12,13,14,15]. For instance, TLR2 signaling influences glucose metabolism and microbial composition [14,15], while TLR4 signaling in adipose tissue regulates insulin sensitivity and obesity-associated inflammation [16,17]. TLR5 deficiency alters gut microbiota composition and promotes metabolic syndrome [18,19], and endosomal TLR7/9 activation has been implicated in autoimmune pathogenesis through aberrant self-nucleic acid recognition [20]. The functional repertoire of TLRs thus extends far beyond innate immune defense, encompassing fundamental physiological processes which are currently being gradually elucidated.

**Figure 1:**
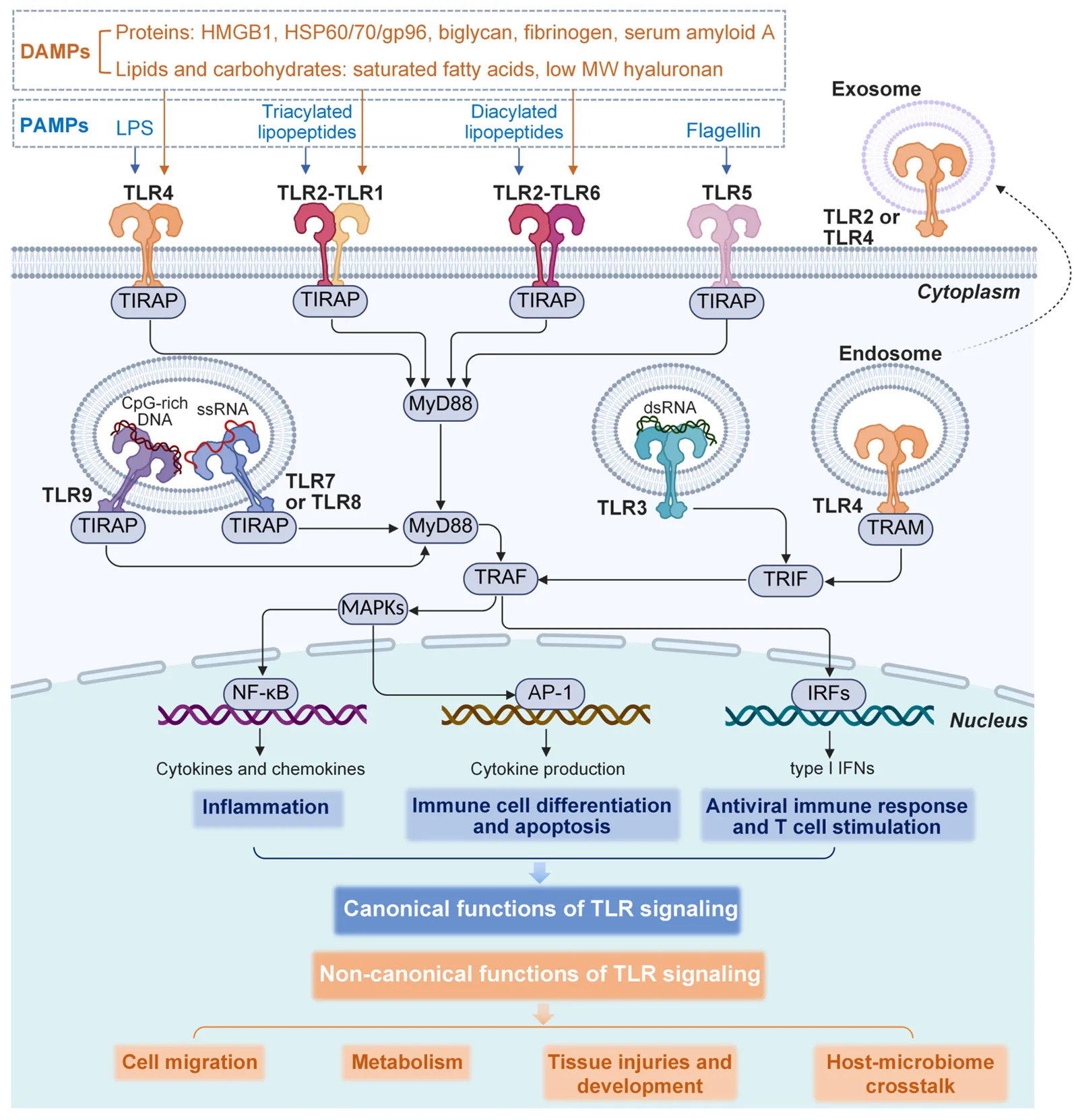
**Schematic representation of Toll-like receptor (TLR) signaling pathways and their biological functions.** TLRs are located either on the plasma membrane (TLR2/1, TLR2/6, TLR4, TLR5) or within endosomes (TLR3, TLR7/8, TLR9). The mammalian TLR family comprises ten functional members (TLR1-TLR10) in humans and twelve (TLR1-TLR9, TLR11-TLR13) in mice. Zebrafish possess orthologs of mammalian TLR1-TLR5, TLR7, TLR8, and TLR9 but also carry fish-specific TLRs (e.g., TLR18, TLR19, TLR22) that are absent in mammals. Conversely, zebrafish lack clear orthologs of mammalian TLR6 and TLR10 [27,28]. Although the TLR systems in zebrafish and mammals are not entirely identical, the core TLR signaling machinery is highly conserved between zebrafish and mammals. Orthologs of nearly all mammalian TLR signaling components, including MyD88, TIRAP/MAL, TRIF/TICAM1, Tollip, TRAF, etc., have been identified in zebrafish, with clear orthologous relationships, including conservation of intron positions, for most core signal transduction components [29,30]. TLRs recognize a diverse array of pathogen-associated molecular patterns (PAMPs), including lipopolysaccharide (LPS), lipopeptides, and flagellin. They also recognize host-derived damage-associated molecular patterns (DAMPs), including high-mobility group box 1 (HMGB1), heat shock proteins (HSPs), and saturated fatty acids and low-molecular-weight (MW) hyaluronan. Upon ligand binding, TLRs recruit adaptor proteins, such as TIRAP and TRAM, to initiate downstream signaling cascades. Most TLRs signal through the MyD88-dependent pathway (except TLR3), leading to the activation of MAPKs and transcription factors NF-κB and AP-1, which drive the production of proinflammatory cytokines. Alternatively, TLR3 and TLR4 utilize the TRIF-dependent pathway to activate IRFs, inducing type I interferons (IFNs) [7,8]. These pathways ultimately regulate canonical functions, such as inflammation, immune cell differentiation and apoptosis, and antiviral responses, as well as non-canonical functions, including cell migration, metabolism, tissue injury/development, and host–microbiome crosstalk.

Upon recognizing PAMPs and DAMPs, TLRs engage specific adaptor proteins to trigger downstream signaling cascades. The Toll-interleukin-1 receptor (TIR) domain-containing adaptor protein (TIRAP), also known as MyD88-associated ligand (MAL), functions as an adaptor protein of TLR2 or TLR4 [21]. As a key intracellular signaling transducer, TIRAP interacts with the TIR domain of TLR2 and subsequently recruits MyD88 [22]. TIRAP thereby initiates the downstream cascade of MyD88-dependent TLR signaling that drives proinflammatory cytokine production and innate immune activation [23,24]. Beyond this well-established role in host defense, emerging evidence suggests that components of the TLR signaling pathway also participate in broader physiological processes. In zebrafish studies, it was shown that *tirap* deficiency not only impairs TLR2-mediated immune signaling but also alters metabolic regulation and enhances neutrophil recruitment velocity following wounding, highlighting its specialized and pleiotropic functions beyond microbial recognition [25]. MyD88 has recently been shown to limit fibrosis during zebrafish heart regeneration by promoting neutrophil recruitment and endocardial PI3K/AKT signaling [26]. In this review, we use the term canonical to refer to the classical, well-established functions of TLRs in immunity, specifically the recognition of PAMPs and DAMPs, activation of MyD88- and/or TRIF-dependent signaling cascades, and the consequent induction of NF-κB-, AP-1-, and IRF-mediated transcriptional programs that drive inflammation, antimicrobial responses, and adaptive immune activation. In contrast, we use non-canonical to refer to TLR functions that operate outside the context of host defense against infection, including the regulation of cell migration, the control of systemic metabolic homeostasis, participation in tissue development, and the shaping of commensal microbial communities (Figure 1). By integrating recent advances across the TLR family, we aim to highlight the paradigm shift from viewing TLRs solely as immune sentinels to recognizing them as pleiotropic regulators of organismal physiology and development.

## 2. The Roles of TLR Signaling in Cell Migration

Recent studies in mammalian and zebrafish systems have shown that individual TLRs exhibit both shared and distinct roles in orchestrating cell dynamics across diverse cellular contexts (Table 1). TLR2 plays a critical role in regulating cell migration across diverse cell types and pathological contexts, operating through both canonical inflammatory signaling and specialized mechanisms involving adaptor proteins and inter-pathway crosstalk [31,32,33,34]. TLR2 signaling exerts context-dependent effects on leukocyte migration. During severe polymicrobial sepsis, TLR2 impairs neutrophil migration by upregulating G-protein-coupled receptor kinase 2 (GRK2), which downregulates CXCR2 expression, thereby reducing chemotaxis, bacterial clearance, and survival [31]. This finding highlights a context-dependent immunosuppressive role of TLR2 that contributes to sepsis pathophysiology. Recent studies utilizing zebrafish models have provided direct in vivo evidence for the role of TLR2 in immune cell dynamics after mycobacterial gut infection [32]. Specifically, TLR2 signaling protects against bacterial proliferation and systemic dissemination and is indispensable for sustaining normal macrophage motility upon infection [32]. In a murine intestinal organoid model, TLR2 blockade using specific antagonist MMG-11 and CU-CPT22 significantly impaired macrophage directional migration towards *Mycobacterium avium*-infected intestinal organoids, as evidenced by reduced migration displacement and mean migratory speed [33]. These findings establish that TLR2 signaling is indispensable for guiding macrophage recruitment to sites of intestinal mycobacterial infection. The TLR2 adaptor protein TIRAP also exerts specialized roles in leukocyte migration. In zebrafish tail-wounding models, *tirap* mutant larvae recruited significantly more neutrophils to wounds with increased migration speed, whereas *tlr2* and *myd88* mutant larvae recruited fewer neutrophils and macrophages [25,34]. This indicates that individual components of the TLR2 signaling cascade can exert distinct and non-redundant effects on cell migration.

Canonical TLR4 signaling enhances neutrophil chemotactic migration by downregulating G protein-coupled receptor kinases GRK2 and GRK5, thereby attenuating chemokine receptor desensitization [35]. In acute lung injury models, TLR4-deficient mice exhibit markedly reduced LPS-induced neutrophil emigration into the alveolar space, accompanied by decreased expression of proinflammatory cytokines, including TNF-α, KC, and CXCL10, confirming that TLR4-mediated inflammatory cascades are essential for neutrophil recruitment [36]. Recent studies have further expanded the mechanistic understanding of TLR4-regulated migration [37,38]. Extracellular vimentin (eVim) and its citrullinated form (CitVim) act as endogenous TLR4 ligands on neutrophils, enhancing adhesion and transendothelial migration [37]. CKLF-like MARVEL transmembrane domain containing (CMTM)3, a TLR4 modulator upregulated in sepsis, promotes neutrophil egress from bone marrow by increasing surface TLR4 and CXCR2 expression. CMTM3 knockout reduces TLR4 levels, attenuates neutrophil migration, and improves survival with less organ damage in septic mice [38].

TLR7/8 signaling also contributes to immune cell dynamics through distinct mechanisms. In vivo studies in rats and mice demonstrated that oral administration of the TLR7/8 ligand R-848 induced a dramatic 20- to 30-fold increase in intestinal dendritic cell output within 10 h, resulting from a near-total release of lamina propria dendritic cells into lymph and subsequent accumulation in mesenteric lymph nodes [39]. These findings illustrate the complex, multi-layered regulation of cell migration by TLR7/8 signaling, involving both direct effects on target cells and indirect effects through intermediary cytokine-producing cells.

Across these studies, a unifying principle emerges, which is that TLR-driven migration is determined less by receptor identity than by which downstream route is engaged in a given cell type. For example, TLR2 and TLR4 can produce opposite migratory outcomes despite sharing TIRAP-MyD88, reflecting differences in G-protein-coupled receptor kinase isoform usage and chemokine receptor turnover [31,35]. Three potential mechanistically distinct routes can be hypothesized: (i) transcriptional control of chemokine receptor availability (ii) direct modulation of adhesion and cytoskeletal machinery, and (iii) indirect effects through intermediary cytokine-producing cells [31,35,36,37,38,39]. These routes are not mutually exclusive, and the same TLR could engage different routes depending on context, underscoring the need to define TLR function by its cellular and physiological setting rather than by receptor identity alone.

## 3. Roles of TLR Signaling in Metabolism

### 3.1. TLR Signaling in Diabetes and Related Metabolic Disorders

While TLRs and their adaptor proteins are classically studied in the context of microbial recognition and innate immune activation, increasing evidence points to their additional roles in regulating metabolic processes (Table 1). In mammalian systems, TLR2 deficiency protects against high fat diet (HFD)-induced inflammation, insulin resistance, hepatic steatosis, and β-cell dysfunction in mice models, suggesting that TLR2 functions at the interface between immune signaling and metabolic regulation [40,41,42,43]. Consistent with these findings, TLR2 expression is elevated in adipose tissue of obese individuals and correlates with inflammatory status and insulin resistance, further supporting its contribution to immunometabolic dysfunction in humans [44]. TLR4 mediates obesity-associated metabolic inflammation by regulating adipogenesis, thermogenesis, and glucose metabolism via endotoxin-driven chronic low-grade inflammation, contributing to the development of type 2 diabetes [16]. In the liver, hepatocyte-specific TLR4 deletion ameliorates hepatic steatosis, inflammation, and insulin resistance in mice fed a high-fat diet [45]. Conversely, TLR5 deficiency in mice leads to altered gut microbiota composition, hyperphagia, and features of metabolic syndrome including obesity and insulin resistance, while administration of flagellin (TLR5 ligand) exerts protective effects against diet-induced obesity [46]. These observations indicate that TLRs are not merely sensors of microbial ligands but also important regulators of metabolic homeostasis, with distinct family members exerting divergent effects on diabetes pathogenesis.

The adaptor protein MyD88, which mediates signaling downstream of most TLRs, plays a central role in integrating immune and metabolic pathways. Mice lacking MyD88 specifically in intestinal epithelial cells partially protected against diet-induced obesity, type 2 diabetes, and inflammation, accompanied by higher energy expenditure [47]. CNS-restricted MyD88 deletion protects mice from high-fat diet-induced leptin resistance and weight gain, defining neuronal MyD88-dependent signaling as a key regulator of diet-induced metabolic dysfunction [48]. In contrast, hepatocyte-specific MyD88 deletion unexpectedly predisposes to hepatic inflammation and insulin resistance even without high-fat diet feeding, suggesting that the role of MyD88 signaling is tissue-dependent, proinflammatory in immune cells but potentially anti-inflammatory in hepatocytes [49]. Collectively, these findings highlight the context-specific nature of MyD88 function and further validate the therapeutic potential of targeting this signaling axis in obesity, as recently demonstrated by the small-molecule MyD88 inhibitor TJ-M2010-5, which attenuates body weight, blood glucose, and lipid levels in high-fat diet mice by suppressing M1 macrophage polarization and metabolic inflammation via the TLR4/MyD88 pathway [50]. Consistently, a cell-penetrating peptide from the MyD88 death domain has been shown to attenuate inflammation and insulin resistance while improving glucose metabolism [51], and MyD88 inhibition through adipose IL-10 induction ameliorates diabetes-induced hepatic inflammation and gluconeogenesis [52]. These studies indicate that the metabolic output of TLR signaling is determined by tissue and cell type rather than by receptor identity.

In the context of diabetic complications, TLR2 and TLR4 signaling have emerged as key drivers of vascular endothelial dysfunction. High glucose promotes neutrophil extracellular trap (NET) formation through TLR2 and TLR4 activation, which in turn enhances neutrophil–endothelial adhesion, inhibits endothelial cell migration, and compromises barrier function; these effects can be partially mitigated by TLR2 and TLR4 antagonists [53]. In diabetic kidney disease, TLR2 and TLR4 stress signaling interacts with sodium–glucose cotransporter-2 (SGLT2) and ER stress pathways, with SGLT2 inhibitors showing preclinical efficacy against TLR2/4 signaling [54]. Wogonin, a natural flavonoid, alleviates diabetic nephropathy injury by upregulating SOCS3 to inhibit TLR4-mediated JAK/STAT/AIM2 signaling [55], and the IDO-1 inhibitor 3-047 combined with icaritin improves diabetes-associated cognitive dysfunction via suppression of the TLR4/MyD88/NF-κB pathway [56]. Microbial metabolites such as trimethylamine (TMA) have been shown to blunt TLR4 signaling by inhibiting IRAK4, decoupling inflammation and insulin resistance from diet-induced obesity and improving glycemic control [57]. These findings indicate that diabetic complications across multiple organ systems converge predominantly on TLR2 and TLR4 signaling, and that this axis is amenable to pharmacological and microbial modulation.

### 3.2. TLR Metabolic Functions in Autoimmune, Endocrine, and Neural Contexts

The metabolic functions of TLRs extend beyond classical metabolic organs to encompass a wide range of physiological and pathological contexts. Extending these findings to non-obese, non-diabetic contexts, a recent study in experimental osteoarthritis demonstrated that pathological upregulation of Tlr2 and Tlr4 expression in cartilage drives NF-κB-mediated inflammation and disrupts cartilage metabolism, while probiotic and chondroitin sulfate supplementation downregulated Tlr2/Tlr4 expression and restored cartilage metabolic markers, further illustrating the translational potential of modulating TLR2/4 signaling in metabolic and inflammatory joint diseases [58]. In rheumatoid arthritis patients, TLR2 is upregulated on CD4^+^ T cells and reprograms glucose metabolism toward the pentose phosphate pathway, driving ROS-mediated cell hyperactivation and TNF-α secretion, indicating that TLR2-dependent metabolic rewiring contributes to autoimmune pathogenesis [59]. Furthermore, thyroid-stimulating hormone (TSH) has been shown to regulate hepatocellular glucose metabolism via the TLR4/Tollip pathway, revealing an endocrine–immune–metabolic axis that may link thyroid dysfunction to metabolic disorders [60]. In dendritic cells, both TLR and C-type lectin receptor agonists induce acute glycolysis that is dependent on glycogen metabolism, and glycogenolysis supports DC maturation, inflammatory cytokine production, and NLRP3 inflammasome priming, demonstrating that TLR-driven metabolic reprogramming is a fundamental requirement for innate immune effector function [61]. Genetic association studies in healthy children and adults have further linked polymorphisms in TLR2, TLR4, and NLR genes to altered lipid and glucose parameters, providing population-level evidence that TLR variants may influence metabolic health [62]. The role of TLR2 in metabolism also extends beyond the immune system to the central nervous system. Using [^18^F] FDG-PET-CT in a mouse model of cardiac arrest and resuscitation, TLR2 deficiency was associated with significantly higher baseline brain glucose uptake and a blunted post-ischemic glucose response, suggesting that TLR2 signaling influences cerebral glucose metabolism under both physiological and ischemic conditions [63]. Together, these studies indicate that TLR-driven metabolic reprogramming operates across joint disease, autoimmune, endocrine, and neural contexts, extending the influence of TLR signaling beyond classical metabolic organs and underscoring its broad role as a context-dependent regulator of cellular metabolism.

### 3.3. Adaptor Proteins Within the Same Pathway Exert Non-Redundant Metabolic Effects

Recent work in zebrafish has extended these findings by demonstrating that TLR signaling also contributes to metabolic regulation during vertebrate development under unchallenged conditions [14,25]. Transcriptomic and metabolomic analyses revealed that *tlr2* mutant zebrafish larvae exhibit altered expression of genes involved in metabolic pathways together with elevated glucose levels, indicating a central role of TLR2 in maintaining glucose homeostasis through transcriptional regulation of metabolic enzymes [14]. Complementary studies on *tirap* and *myd88* mutant zebrafish larvae further highlight that adaptor proteins within the TLR signaling pathway can exert distinct and non-redundant metabolic effects. In particular, *tirap* mutant larvae display reduced glucose concentrations and differential regulation of pathways related to calcium homeostasis and myosin-associated functions, whereas *myd88* mutation does not significantly affect glucose levels [25]. These findings are especially notable because they suggest that different components of the TLR signaling cascade do not contribute equally to metabolic regulation, revealing an additional layer of functional complexity within the pathway.

Taken together, these studies support an emerging view that TLR signaling extends well beyond its classical role in host defense. By linking innate immune signaling with glucose regulation, lipid metabolism, cellular homeostasis, and broader metabolic pathways, TLRs appear to function as important integrators of immunological and metabolic states. The diversity of metabolic phenotypes observed across different TLR mutants indicates that individual TLRs have both shared and distinct contributions to metabolic regulation, likely reflecting their unique ligand specificities, cell-type expression profiles, and downstream signaling repertoires. In the context of diabetes, TLR2 and TLR4 activation by fatty acids and microbial products drives chronic low-grade inflammation, disrupts insulin signaling in peripheral tissues, and promotes β-cell dysfunction [16,45]. TLR5 deficiency illustrates how altered microbial sensing can predispose to metabolic syndrome [46], while MyD88-dependent signaling in different tissues orchestrates systemic responses to nutritional excess [47,48,49]. These mechanistic insights position TLR signaling as a promising therapeutic target for preventing or treating diabetes and related metabolic diseases, with strategies ranging from TLR antagonists to MyD88 inhibitors currently under investigation.

## 4. Roles of TLR Signaling in Tissue Injury and Development

### 4.1. TLR Signaling in Tissue Injury and Repair

TLRs are constitutively expressed in non-immune tissues and play critical roles in responses to sterile injury (Table 1). In the kidney, TLR2 protein is constitutively expressed in renal tubules, glomeruli, and vasculature, and TLR2 deficiency confers protection against ischemic renal injury through both MyD88-dependent and -independent pathways [64]. Notably, TLR2-deficient mice were better protected than MyD88-deficient mice, raising the possibility that TLR2 signals through alternative adaptors such as TRIF to mediate kidney injury [64]. This constitutive expression pattern suggests that TLR2 may serve as a sentinel for tissue damage even in the absence of infection.

In the heart, TLR2 signaling modulates left ventricular function following ischemia–reperfusion injury [65]. TLR2-deficient mouse hearts show significantly better recovery of contractile performance compared to wild-type controls, associated with reduced cardiac levels of TNF-α and IL-1β, and ischemia–reperfusion-induced left ventricular dysfunction was reversed by TNF blockade [65]. Recent findings demonstrate that MyD88 limits fibrosis during zebrafish heart regeneration by promoting neutrophil recruitment and endocardial PI3K/AKT signaling [26]. TLR4 also participates in vascular remodeling processes. In a femoral artery cuff model, TLR4 activation by LPS stimulated plaque formation and outward arterial remodeling, whereas TLR4-deficient mice showed no outward remodeling despite neointima formation [66]. Similarly, following carotid artery ligation, wild-type mice exhibited outward remodeling associated with increased TLR4 expression and endogenous TLR4 ligands (EDA and Hsp 60), while TLR4-deficient mice did not [66]. This provides genetic evidence that TLR4 is required for outward arterial remodeling, a process critical for vascular adaptation to hemodynamic changes. Together, these studies illustrate both the detrimental and protective roles of TLR/MyD88 signaling in cardiac injury versus repair, depending on context and timing.

Arteriogenesis, the growth of collateral arteries, also requires TLR2 and TLR4 expression in bone marrow-derived cells. TLR2-null and TLR4-defective mice showed significantly reduced collateral perfusion following femoral artery occlusion [67]. Bone marrow transplantation experiments revealed that wild-type bone marrow transplanted into TLR-deficient hosts rescued impaired arteriogenesis, while TLR-deficient bone marrow transplanted into wild-type hosts reduced collateral growth to TLR-deficient levels [67]. This demonstrates that TLR2 and TLR4 expression by infiltrating leukocytes, rather than by vascular cells, is essential for adaptive collateral artery growth.

Beyond the heart and vasculature, the injury-related functions of TLRs are particularly prominent in the nervous system. Following ischemic stroke, TLR2 and TLR4 expressions are elevated in cerebral cortical neurons, and mice deficient in either receptor exhibit significantly reduced brain damage and neurological deficits [68]. These findings establish a proapoptotic signaling pathway for TLR2 and TLR4 in neurons, revealing that TLRs can directly influence neuronal survival during ischemic injury.

### 4.2. TLR Signaling in Development and Neural Tissue Organization

TLR signaling has also been implicated in diverse aspects of development, including neural differentiation and brain tissue architecture (Table 1) [69,70]. Early research in Drosophila established Toll signaling as a key determinant of dorsoventral axis formation, providing the first link between the function of TLRs in embryonic patterning and their later discovered function as innate immune receptors [71]. This historical progression has fundamentally broadened the conceptual framework of TLR biology, suggesting that these receptors are not restricted to host defense but may also participate in developmental programs that shape tissue organization and cellular identity. Such functions point to a broader role for TLR signaling in coordinating developmental and homeostatic processes across multiple organ systems.

Studies in vertebrates have shown that TLRs regulate processes such as neurogenesis, axonal guidance and synaptic plasticity [69,72,73]. These observations support the view that TLR signaling contributes to the establishment and maintenance of the nervous system, possibly by integrating environmental cues with intrinsic developmental pathways. In this context, TLRs may influence not only immune surveillance within developing tissues but also the behavior of neural progenitor cells, neuronal connectivity, and structural remodeling of the brain. In the brain, neurons express multiple TLRs, with TLR2 and TLR4 levels increased upon IFN-γ stimulation and energy deprivation. Neurons from TLR2- and TLR4-deficient mice are protected against energy deprivation-induced cell death through reduced JNK and AP-1 proapoptotic signaling [68]. Recent evidence from zebrafish models provides compelling in vivo support for developmental functions of TLR2. Using ultrahigh-field diffusion magnetic resonance imaging (MRI), striking microstructural alterations are reported in the adult zebrafish brain following *tlr2* genomic deletion, indicating that TLR2 contributes to the maintenance of neural tissue integrity and may participate in neurodevelopmental organization [70]. These findings are particularly significant because they suggest that the developmental functions of TLR2 can have long-lasting consequences that remain detectable in adulthood. Rather than acting solely during acute immune challenge, TLR2 may influence tissue architecture over the course of development, thereby affecting the structural and functional maturation of the nervous system.

Taken together, these studies reveal that TLR signaling plays pleiotropic roles in tissue injury responses and developmental processes across multiple organ systems. In the context of sterile injury (ischemia–reperfusion, stroke, myocardial infarction), TLR2 and TLR4 signaling can be detrimental by promoting excessive inflammation and cell death, yet MyD88-dependent signaling pathways in the heart are required for proper tissue repair and regeneration [26,74,75,76]. In vascular biology, TLR2 and TLR4 are essential for adaptive remodeling and collateral artery growth, highlighting their beneficial roles in maintaining tissue perfusion [66,67]. Further studies, particularly in tractable in vivo systems such as zebrafish, will be important for clarifying how TLR signaling interfaces with developmental pathways and how disruption of this signaling axis may affect organismal development and tissue homeostasis.

## 5. Roles of TLR Signaling in Microbiome Crosstalk

### 5.1. The Microbiome and Host–Microbe Interactions

The microbiome, comprising the diverse communities of microorganisms inhabiting the skin, respiratory tract, gastrointestinal tract, and other mucosal surfaces, is now recognized as an integral component of host biology [77]. Advances in high-throughput sequencing and multi-omics approaches have revealed that the composition and function of these microbial communities are tightly linked to host health and disease [77,78]. Far from being passive residents, commensal microbes engage in dynamic interactions with their hosts, influencing digestion, nutrient metabolism, immune system development, and protection against pathogen colonization [79,80]. Among the microbial habitats within a host, the gut microbiome is the most densely populated and extensively studied [81]. Growing evidence indicates that the microbiome, especially gut microbiota, plays a pivotal role in maintaining host homeostasis, whereas its dysbiosis has been strongly associated with a wide range of metabolic and inflammatory disorders, including obesity, inflammatory bowel disease (IBD) and colorectal cancer (CRC) [82,83]. Therefore, restoration of microbial balance has shown therapeutic promise, underscoring the central role of the gut microbiome as both a determinant of health and a potential target for disease intervention.

### 5.2. The Microbiome and TLR Signaling Interactions

The interactions between multicellular organisms and the microbes surrounding them are based on molecular recognition processes that regulate their responses to each other. On the one hand, the host requires accurate recognition to prevent disease. On the other hand, many microbes are driven to find a suitable environmental niche inside or outside the host for survival. Several studies in mammalian and zebrafish have pointed to the central role of the TLR pathway in many aspects of host sensing of the microbiome [84,85,86,87,88]. Understanding the complex interplay between TLRs and the microbiome requires a detailed knowledge of where and when TLRs are expressed in the intestinal epithelium. A comprehensive map of TLR expression, generated using TLR reporter mice, has revealed unexpected spatial and cell type-specific distribution patterns [89]. Notably, small intestinal epithelial cells express low levels of TLR2, TLR4 and TLR5, whereas they are highly expressed by colonic epithelial cells, indicating a stark regional difference in innate immune sensing capacity along the gastrointestinal tract [89]. Furthermore, TLR5 expression in the small intestine is restricted to Paneth cells in the crypts, whereas colonic epithelial cells express TLR5 more broadly [89]. These findings highlight that the responsiveness of epithelial cells to microbial stimuli is highly dependent on their location and cell identity, a concept with important implications for understanding region-specific host–microbiome interactions in health and disease.

Among gut-expressed TLRs, TLR2 is one of the most extensively studied for its roles in mediating host–microbiome crosstalk (Table 1). TLR2-mediated microbial pattern recognition has been shown to be important for facilitating tolerance to commensal microbial colonization [90], induction of mucin secretion [91] and protection of intestinal barrier integrity in induced inflammation models [92,93]. With the exception of TLR3, all TLRs can signal through the central intracellular adapter MyD88 [28]. Several studies support the importance of TLR signaling for host intestinal immune homeostasis. This includes studies of TLR mutants in a mouse model that were found to develop inflammatory intestinal responses without pathogenic challenge [18,94]. Furthermore, studies in zebrafish larvae found that bacterial signals regulate epithelial cell proliferation in the intestines via MyD88 signaling [95]. It was subsequently shown that microbiota promote MyD88-dependent signaling that inhibits host Notch signaling in the intestinal epithelium, thereby promoting secretory cell fate determination [95]. Another study using a larval zebrafish microbiome model shows that microbiome control of the host transcriptome and macrophage infiltration of the intestines is governed by the TLR signaling downstream adaptor gene *myd88* [85]. TLR2 is confirmed to be essential for microbial control of *myd88* transcription and intestinal immune balance [85]. Therefore, understanding the complex interplay between TLR2 and the microbiome not only reveals fundamental insights into mucosal immunity but also provides potential therapeutic strategies in dysbiosis-related diseases such as IBD. An important functional link has been established between microbiota, TLR2, and regulatory B cells in maintaining colonic homeostasis. Resident enteric bacteria stimulate the development of intestinal IL-10-producing B cells through the TLR2/MyD88/PI3K pathway, and these regulatory B cells, in turn, reduce colonic T cell activation and maintain mucosal homeostasis in response to intestinal microbiota [96]. In addition, the TLR adaptor MyD88 in hematopoietic cells is required for the maintenance of homeostatic innate immune pockets in the intestinal epithelium, where localized increases in bacterial proximity stimulate cytokine production that enhances the immunological barrier [97]. This mechanism highlights how TLR2-mediated microbial sensing directly shapes the regulatory immune landscape in the gut.

Beyond TLR2, emerging evidence highlights the contributions of other TLR family members to host–microbiome crosstalk. TLR4, for instance, plays a critical role in the establishment of gut homeostasis. During early postnatal development in mice, the immunostimulatory activity of the intestinal microflora is largely mediated via TLR4 during breast-feeding, whereas it becomes TLR4-independent after weaning, a transition associated with a shift from a microflora rich in TLR4-stimulatory Proteobacteria to one dominated by Bacteroidales and/or Clostridiales [98]. TLR4-mediated recognition of bacterial LPS by intestinal epithelial cells promotes epithelial cell proliferation, restitution, and protection against apoptosis, contributing to mucosal repair following injury [99,100]. However, dysregulated TLR4 signaling in the intestinal epithelium can promote dysbiosis, increasing susceptibility to colitis and tumorigenesis [100].

TLR5, which recognizes bacterial flagellin, has been specifically implicated in the regulation of intestinal mucosal immunity and barrier function [101,102]. The distribution of TLRs along the gastrointestinal tract is influenced by the composition of commensal residents, and this interaction restricts microbial colonization to particular areas while enabling the gut-associated lymphoid tissue to detect pathogenic invaders [89,102]. TLR9, which recognizes bacterial CpG DNA, has been shown to be important for the balance of regulatory T, Th17, and Th1 cells in the intestine [103].

Therefore, understanding the complex interplay between TLRs and the microbiome not only reveals fundamental insights into mucosal immunity but also provides potential therapeutic strategies in dysbiosis-related diseases such as IBD. Modulating TLR signaling or leveraging TLR–microbiota interactions may emerge as a novel strategy to restore mucosal immune balance and reshape the gut microbiome in IBD patients. However, for this strategy to succeed, it is essential to further understand which features of the normal microbiome are not inherently associated with the onset of pathological symptoms. Consequently, extensive translational studies are needed to explore TLR or microbiome-based interventions as adjunctive therapies aimed at reestablishing intestinal homeostasis and improving disease outcomes.

**Table 1:** **Emerging non-canonical TLR functions across model** **systems.**

**TLR Signaling Members**	**Specific Roles in Cell Migration**	**Cell Types or Tissues**	**Model Systems**
TLR2	Impairs neutrophil chemotaxis in sepsis via GRK-2/CXCR2	Neutrophils	Mouse [31]
	Sustains macrophage motility during mycobacterial gut infection	Macrophages	Zebrafish [32], mouse organoid [33]
TLR4	Enhances neutrophil chemotaxis via GRK2/5 downregulation	Neutrophils	Mouse [35]
	Essential for LPS-induced neutrophil emigration into alveolar space	Neutrophils	Mouse [36]
	Endogenous ligands eVim and CitVim enhance neutrophil adhesion and transendothelial migration	Neutrophils	Mouse [37]
	CMTM3 upregulates surface TLR4 and CXCR2, promoting neutrophil egress from bone marrow	Neutrophils	Mouse [38]
TLR7/8	Induces dendritic cell mobilization and accumulation in mesenteric lymph nodes	Dendritic cells	Rat, mouse [39]
MyD88/TIRAP	Non-redundant roles in leukocyte recruitment	Neutrophils, macrophages	Zebrafish [25,34]
**TLR Signaling Members**	**Specific Roles in Metabolism**	**Cell Types or Tissues**	**Model Systems**
TLR2	Deficiency protects against HFD-induced inflammation, insulin resistance, hepatic steatosis, and β-cell dysfunction	Adipose, liver, β-cells	Mouse [40,41,42,43]
	Expression elevated in adipose tissue of obese individuals	Adipose	Human [44]
	Upregulated in CD4^+^ T cells and reprograms glucose metabolism	CD4^+^ T cells	Human [59]
	Deficiency associated with higher baseline brain glucose uptake and blunted post-ischemic glucose response	Brain	Mouse [63]
	*tlr2* mutant larvae exhibit altered metabolic gene expression and elevated glucose levels	Whole larvae	Zebrafish [14]
TIRAP	*tirap* mutant larvae display reduced glucose concentrations and altered calcium/myosin-associated pathways	Whole larvae	Zebrafish [25]
TLR4	Mediates obesity-associated metabolic inflammation by regulating adipogenesis, thermogenesis, and glucose metabolism	Adipose	Mouse [16]
	Hepatocyte-specific deletion ameliorates hepatic steatosis, inflammation, and insulin resistance	Liver, hepatocytes	Mouse [45]
	Wogonin alleviates diabetic nephropathy by upregulating SOCS3 to inhibit TLR4-mediated JAK/STAT/AIM2 signaling	Kidney	Mouse [55]
	IDO-1 inhibitor 3-047 combined with icaritin improves diabetes-associated cognitive dysfunction via suppression of TLR4/MyD88/NF-κB	Hippocampus, microglia	Mouse [56]
	Microbial metabolite TMA blunts TLR4 signaling by inhibiting IRAK4, decoupling inflammation and insulin resistance from diet-induced obesity	Liver	Mouse [57]
	TSH regulates hepatocellular glucose metabolism via TLR4/Tollip pathway	Liver, hepatocytes	Mouse [60]
TLR2/TLR4	High glucose promotes neutrophil extracellular trap (NET) formation through TLR2 and TLR4 activation	Vascular endothelium	Mouse [53]
	TLR2/4 stress signaling interacts with sodium–glucose cotransporter-2 (SGLT2) and ER stress pathways	Kidney	Mouse [54]
	Pathological upregulation in cartilage drives NF-κB-mediated inflammation and disrupts cartilage metabolism	Cartilage, joint	Mouse [58]
	Genetic polymorphisms linked to altered lipid and glucose parameters	Systemic	Human [62]
TLR5	Deficiency causes hyperphagia and promotes metabolic syndrome (obesity, insulin resistance)	Systemic	Mouse [46]
MyD88	Deficiency in intestinal epithelial cells partially protected against diet-induced obesity	Intestinal epithelium	Mouse [47]
	CNS-restricted deletion protects against HFD-induced leptin resistance and weight gain	CNS	Mouse [48]
	Hepatocyte-specific deletion predisposes to hepatic inflammation and insulin resistance even without HFD	Liver, hepatocytes	Mouse [49]
	Small-molecule inhibitor TJ-M2010-5 attenuates body weight, blood glucose, and lipid levels	Bone marrow-derived macrophages, adipose, liver	Mouse [50]
	Cell-penetrating peptide from MyD88 death domain attenuates inflammation and insulin resistance	Peritoneal macrophages, skeletal muscle	Rat, mouse [51]
	Inhibition ameliorates diabetes-induced hepatic inflammation and gluconeogenesis	Adipose, liver	Mouse [52]
**TLR Signaling Members**	**Specific Roles in Tissue Development and Injury**	**Cell Types or Tissues**	**Model Systems**
TLR2	Mediates ischemic renal injury via MyD88-dependent and -independent pathways	Kidney	Mouse [64]
	Modulates left ventricular function following ischemia–reperfusion injury	Heart	Mouse [65]
	Deletion causes long-lasting microstructural alterations in adult brain	Brain	Zebrafish [70]
TLR4	Required for outward arterial remodeling	Vascular smooth muscle	Mouse [66]
TLR2/TLR4	Required for arteriogenesis in bone marrow-derived cells	Collateral arteries	Mouse [67]
MyD88	Limits fibrosis during heart regeneration by promoting neutrophil recruitment and endocardial PI3K/AKT signaling	Endocardium, neutrophils	Zebrafish [26]
**TLR Signaling Members**	**Specific Roles in Microbiome Crosstalk**	**Cell Types or Tissues**	**Model Systems**
TLR2	Facilitates tolerance to commensals, induces mucin secretion, and protects barrier integrity	Intestinal epithelium	Mouse [90,91,92,93]
	Essential for microbial control of *myd88* transcription and intestinal immune balance	Intestinal epithelium	Zebrafish [85]
	Resident enteric bacteria stimulate IL-10-producing regulatory B cells via TLR2/MyD88/PI3K pathway	B cells, colon	Mouse [96]
	Influences microbial diversity and community composition in a development-dependent manner	Gut	Zebrafish [15]
TLR4	Mediates immunostimulatory activity of intestinal microflora during breast-feeding	Intestinal epithelium	Mouse [98]
	Recognizes bacterial LPS to promote epithelial proliferation, restitution, and protection against apoptosis, contributing to mucosal repair	Intestinal epithelium	Mouse [99,100]
	Dysregulated signaling promotes dysbiosis, increasing susceptibility to colitis and tumorigenesis	Intestinal epithelium	Mouse [100]
TLR5	Recognizes bacterial flagellin, regulates intestinal mucosal immunity and barrier function	Intestinal epithelium	Mouse [101]
TLR9	Recognizes bacterial CpG DNA; important for balance of regulatory T, Th17, and Th1 cells in intestine	T cells/intestine	Mouse [103]
MyD88	Governs microbiome control of host transcriptome and macrophage infiltration of the intestines	Intestinal epithelium, macrophages	Zebrafish [85]
	Microbiota promote MyD88-dependent signaling that inhibits Notch signaling, promoting secretory cell fate determination	Intestinal epithelium	Zebrafish [95]
	Required in hematopoietic cells for maintenance of homeostatic innate immune pockets in intestinal epithelium	Hematopoietic cells, intestinal epithelium	Mouse [97]

## 6. Zebrafish and Organoid Model Systems for Studying the Systemic Functions of TLR Signaling

### 6.1. Zebrafish Models

Although the TLR systems in zebrafish and mammals are not entirely identical, the core signaling mechanisms (adaptor usage, kinase cascades, NF-κB/IRF activation) are sufficiently conserved to justify using zebrafish to dissect TLR pathway function relevant to mammals (Figure 1) [27,28,29,30]. The zebrafish larval model also offers unique advantages for dissecting host–microbe interactions in vivo due to its genetic tractability and amenability to versatile gnotobiotic techniques [84,104]. The zebrafish gut shares many anatomical and physiological features with the human gastrointestinal tract, including conserved epithelial cell types, mucus secretion, innate immune components, and regional specialization [105,106]. Moreover, the zebrafish microbiome exhibits both taxonomic and functional similarities to that of mammals, making it a relevant system for translational microbiome research [105,106]. Importantly, zebrafish larvae can be raised under germ-free (gnotobiotic) conditions and subsequently colonized with defined microbial communities, enabling controlled experimental manipulation of host–microbiome interactions [84,107]. Studies using *tlr2* mutant zebrafish models have shown that TLR2 influences microbial diversity and community composition in a development-dependent manner, suggesting that TLR2 is not only involved in microbial recognition but also plays an important role in shaping host–microbiome interactions during intestinal maturation [15]. These findings further support the idea that TLR2 contributes to the establishment and maintenance of microbial homeostasis across developmental stages.

**Figure 2:**
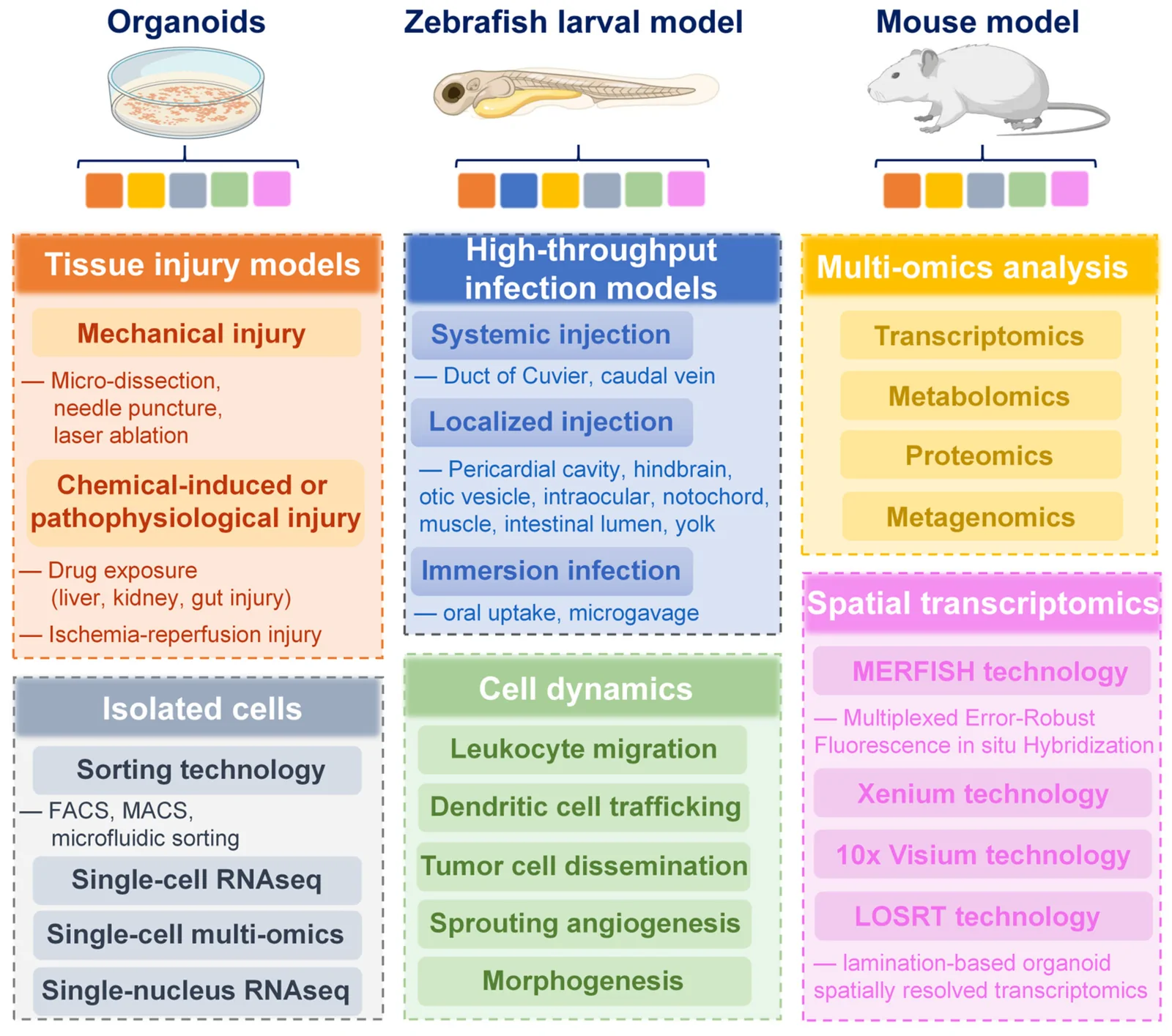
**Overview of experimental approaches across organoid, zebrafish larval, and mouse models.** This schematic summarizes the major experimental platforms and techniques available in organoid, zebrafish larval, and mouse models for studying TLR signaling. Tissue injury can be induced mechanically, chemically or pathophysiologically [25,34]. High-throughput infection models include systemic and localized microinjection as well as immersion-based routes [32,111]. Cell-sorting technologies can be categorized into fluorescence-activated cell sorting (FACS), magnetic-activated cell sorting (MACS), and microfluidics-based physical methods. Isolated cells can be subjected to single-cell RNA-seq, single-cell multi-omics, and single-nucleus RNA-seq [117,118]. Cell dynamics encompass leukocyte migration, dendritic cell trafficking, tumor cell dissemination, sprouting angiogenesis, and morphogenesis [119]. Spatial transcriptomics technologies, including MERFISH (Multiplexed Error-Robust Fluorescence in situ Hybridization), Xenium, 10x Visium, and LOSRT (lamination-based organoid spatially resolved transcriptomics), enable the mapping of gene expression within tissue architecture, bridging cellular resolution with spatial context [120,121,122]. Tissue injury (presented in orange area), multi-omics analyses (yellow area), isolated cells (grey area), cell dynamics (green area) and spatial transcriptomics (pink area) can be studied across organoid, zebrafish larval, and mouse models, while high-throughput infection models (blue area) are primarily enabled by zebrafish models [123].

The zebrafish model also facilitates genetic dissection of host pathways through CRISPR/Cas9-based genome editing approaches such as CRISPRANTs, which enable rapid generation of targeted mutants in F0 larvae [108,109]. In addition, recent advances in robotic microinjection platforms in zebrafish larvae have increased experimental throughput and reproducibility. Automated platforms now permit high-precision delivery of bacteria into specific sites such as the Duct of Cuvier, allowing standardized infection assays [110,111]. In parallel, using a robotic microinjection system, automated and precise delivery of bacteria or microbial products into gut lumen also becomes possible, allowing standardized infection and colonization assays [32]. By combining real-time imaging of immune responses, controlled microbial colonization, and scalable genome editing and automated microinjection technologies, zebrafish provide a powerful system to investigate the interplay between the microbiome and host immunity (Figure 2). In addition, single cell transcriptomic and spatial transcriptomic approaches linked to whole organism transcriptome databases have been implemented successfully in zebrafish larval studies (Figure 2). Collectively, these technical capabilities establish zebrafish larvae as a uniquely versatile platform for dissecting the cellular and molecular mechanisms underlying host–microbiome interactions that is poised to accelerate the translation of fundamental discoveries into therapeutic strategies for microbiome-associated diseases.

### 6.2. Mammalian Organoid Models

Human and mouse organoids have emerged as powerful complementary systems to whole test animals for dissecting cellular signaling in tissue-specific contexts (Figure 2). For TLR signaling, in the gastrointestinal tract, human and murine organoid-based studies have revealed that TLR expression patterns are largely determined during development and retained in tissue-resident stem cells, with TLR4 expression restricted to the stomach and colon but absent in the small intestine in a microbiota-independent manner [112]. Functional assays have shown that gastric organoids respond to LPS via TLR4/NF-κB signaling, whereas small intestinal organoids remain refractory to TLR2 and TLR4 agonists, a finding that underscores the importance of spatial organization and species specificity in epithelial innate immunity [112]. In human fetal intestinal organoids (FEnS), treatment with the TLR4 inhibitor TAK242 increased transepithelial permeability, confirming a functional role for TLR4 in fetal intestinal epithelial barrier maintenance and cell proliferation [113]. In lung organoids, TLR4/NF-κB activation has been implicated in host defense against *Mycobacterium tuberculosis* [114], while TLR3-mutant patient-derived organoids have provided mechanistic insights into severe COVID-19 [115]. In cerebral organoids containing microglia, TLR3 activation induces type I/III interferons and antiviral factors against HIV-1, demonstrating that organoid models can recapitulate complex neuroimmune interactions [116]. Recently, mouse ileal organoids have been used as a model for studying infection by mycobacteria [33]. Together with zebrafish studies, these organoid-based approaches provide a multi-model framework for understanding how TLR signaling integrates with tissue-specific and species-specific cues to shape physiological and pathological outcomes. The zebrafish offers unparalleled advantages for studying dynamic immune cell behavior, microbial colonization, and developmental processes in a live vertebrate context, while mammalian organoids enable mechanistic dissection in genetically defined human cell types under controlled conditions. Moving forward, the integration of zebrafish in vivo dynamics with organoid-based human cellular platforms will be key to unraveling the context-dependent roles of TLR signaling across scales, from molecules to tissues to organisms.

## 7. TLR Agonists and Antagonists in Clinical Development

The dual nature of TLR signaling, protective when properly regulated but pathogenic when dysregulated, has positioned these receptors as attractive therapeutic targets [124,125]. The consensus view is that TLR agonists can serve as therapeutic agents or vaccine adjuvants for cancers and infectious diseases, while TLR antagonists may offer a promising approach to treating autoimmune diseases and certain inflammatory conditions [126,127]. While the current literature heavily emphasizes TLR-targeting agents in oncology, an expanding body of evidence points to their considerable potential across a broader spectrum of non-malignant diseases, including autoimmune disorders, chronic inflammatory conditions, and infections [126,128]. Several recent reviews have summarized up-to-date tabulations of TLR modulator clinical trials, including detailed information on trial phase, sample size, and endpoint results [126,129,130]. Here, we focus on summarizing the development potential of TLR modulators beyond cancer, highlighting emerging clinical applications in other disease areas.

### 7.1. TLR Agonists in Infectious Diseases and Vaccines

Beyond their established role in cancer immunotherapy, TLR agonists are being extensively investigated as vaccine adjuvants and direct anti-infective agents [126]. For example, in squalene-based emulsion formulations, TLR2/1 agonist Pam3CSK4 enhances protective antibody responses to polysaccharide vaccines in both mouse and humanized models [131]. TLR4 agonist GLA-SE adjuvanticity acts through TLR4 signaling and NLRP3 inflammasome activation, with the IL-1β-dependent axis serving as a central effector that amplifies antigen-specific CD4^+^ T and B cell responses [132]. In the context of respiratory infections, TLR agonists such as PUL-042, a TLR2/6 and TLR9 agonist, are being evaluated in clinical trials for their potential to enhance host defense against bacterial and viral pathogens [133,134]. In chronic hepatitis B, TLR8 agonists such as selgantolimod and TQA3810 are currently in clinical trials, aiming to restore antiviral immunity in chronically infected patients [135].

### 7.2. TLR Antagonists in Autoimmune and Inflammatory Diseases

The most significant non-oncology advance in TLR-targeted therapy has been in autoimmune diseases, particularly systemic lupus erythematosus (SLE) and cutaneous lupus erythematosus (CLE). Endosomal TLR7 and TLR8 have emerged as key pathogenic drivers in lupus, where aberrant recognition of self-nucleic acids triggers type I interferon production and autoantibody generation [135,136,137]. The oral dual TLR7/8 antagonist enpatoran demonstrated a significant dose-dependent effect on disease activity in patients with CLE or SLE with active skin manifestations [136,137]. Beyond lupus, TLR antagonists are being explored in psoriasis: the novel small-molecule TLR7/8 antagonist SMU-R39, developed through structure-based drug design from an agonist scaffold, has demonstrated efficacy in ameliorating psoriasis-like skin inflammation in mouse models without inducing systemic toxicity [138].

Beyond systemic autoimmunity, the therapeutic application of TLR antagonists is being increasingly explored in chronic, progressive inflammatory diseases closely linked to microbial dysbiosis, such as IBD. In IBD, aberrant TLR activation by commensal or pathogenic microbiota is a sustained driver of intestinal inflammation [139]. Preclinical studies have demonstrated that TLR4 antagonists such as CRX-526 and 1A6 can block acute inflammatory infiltrate and cytokine responses in murine colitis models, while TLR9 antagonists (AV-ODN) have shown immunomodulatory and tolerance-inducing effects [139]. A cell-penetrating TLR inhibitor peptide (cpTLR-i) derived from the TIR domain of TIRAP has been shown to suppress TNF-α and IL-1β induction in macrophages, ameliorate colonic tissue damage in DSS-induced colitis mice, and promote regulatory macrophage polarization while reducing Th17 responses in the inflamed colonic lamina propria, suggesting the therapeutic potential of TLR inhibition in IBD [140]. As discussed in Section 2, TLR2 antagonists MMG-11/CU-CPT22 impair macrophage migration toward mycobacteria-infected organoids [33]. The link between mycobacteria and chronic intestinal diseases has been a subject of investigation for over a century, dating back to Dalziel’s 1913 observation of pathological similarities between human Crohn’s disease (CD) and Johne’s disease in ruminants caused by *M. avium* subspecies *paratuberculosis* (MAP) [141,142]. While the causal relationship remains debated, current evidence supports a model whereby MAP infection, along with gut microbiome dysbiosis and host genetic susceptibility, may contribute to disease pathogenesis in a subset of IBD patients [143,144]. These preclinical findings suggest that TLR antagonists may offer a broader translational horizon beyond classical autoimmune indications, particularly in chronic inflammatory diseases driven by microbial–immune dysregulation.

## 8. Conclusions

This review has surveyed the expanding landscape of TLR signaling beyond classical immunity, integrating evidence from cell migration, immunometabolism, tissue development and injury, and host–microbiome interactions. This indicates that TLR signaling acts as a context-dependent integrator, whose output is dictated not only by receptor identity alone but also by adaptor usage, cell type, tissue microenvironment, and developmental stage. Looking forward, several key questions remain unresolved. At the molecular level, the precise mechanisms by which different adaptors engage distinct effector programs within the same cell require further dissection. At the cellular level, how TLR-driven metabolic reprogramming is spatially and temporally coordinated with migratory machinery remains poorly understood. At the organismal level, the extent to which TLR–microbiome interactions during early development shape long-term metabolic and immune trajectories is only beginning to be explored. Addressing these requires cross-model integration. Zebrafish larvae enable real-time imaging and high-throughput screening, organoids provide human-relevant mechanistic platforms, and rodents supply systemic context for translational validation. In the near future, we expect that the rapid advances in spatial transcriptomics technologies (Figure 2) will be of great value for better understanding the integrative functions of TLR signaling at the systemic level. Therapeutically, the context-dependent nature of TLR function suggests that selective targeting should be adopted rather than broadly agonistic or antagonistic strategies. These strategies include context-selective modulators, biomarker-driven stratification, and tissue-targeted delivery (e.g., using nanovesicles [145]), thereby translating these insights into safe therapies for infectious, inflammatory, metabolic, and microbiome-associated diseases.

## Data Availability

No new data were created or analyzed in this study.

## Abbreviations

The following abbreviations are used in this manuscript:

TLRs	Toll-like receptors
PRRs	pattern recognition receptors
PAMPs	pathogen-associated molecular patterns
DAMPs	damage-associated molecular patterns
MyD88	myeloid differentiation primary response 88
TIR	Toll/interleukin-1 receptor
TRIF	TIR domain-containing adaptor inducing interferon-β
LPS	lipopolysaccharide
HMGB1	high-mobility group box 1
HSPs	heat shock proteins
TIRAP	TIR domain-containing adaptor protein
MAL	MyD88-associated ligand
PI3K/AKT	phosphoinositide 3-kinase/Protein kinase B
MW	molecular weight
MAPK	mitogen-activated protein kinase
TRAM	TIR domain-containing adaptor molecule
NF-κB	nuclear factor kappa-light-chain-enhancer of activated B
AP-1	activator protein 1
IFNs	type I interferons
IRF	interferon regulatory factor
Pam3CSK4	tripalmitoyl-S-glyceryl-cysteinyl-seryl-(lysyl)_4_
SLE	erythematosus
CLE	cutaneous lupus erythematosus
IBD	inflammatory bowel disease
TNF-α	tumor necrosis factor-alpha
IL-1β	interleukin-1 beta
DSS	dextran sulfate sodium
CD	Crohn’s disease
MAP	*Mycobacteria avium* subspecies *paratuberculosis*
GRK-2	G-protein-coupled receptor kinase-2
CXCR2	C-X-C motif chemokine receptor 2
CXCL10	C-X-C motif chemokine ligand 10
CMTM	CKLF-like MARVEL transmembrane domain containing
eVim	extracellular vimentin
CitVim	citrullinated vimentin
CLL	chronic lymphocytic leukemia
STAT3	signal transducer and activator of transcription 3
CNS	central nervous system
NET	neutrophil extracellular trap
TMA	trimethylamine
IRAK4	interleukin-1 receptor-associated kinase 4
ROS	reactive oxygen species
TSH	thyroid-stimulating hormone
DC	dendritic cell
NLRP3	NLR family pyrin domain-containing 3
NLR	NOD-like receptor
EDA	extra domain A of fibronectin
IFN-γ	interferon-gamma
JNK	c-Jun N-terminal kinase
MRI	magnetic resonance imaging
CRC	colorectal cancer
CpG DNA	unmethylated cytosine-phosphate-guanine dinucleotide-rich DNA
CRISPR	Clustered Regularly Interspaced Short Palindromic Repeats
Cas9	CRISPR-associated protein 9
HIV	human immunodeficiency virus
NTM	nontuberculous mycobacteria
